# Fatty Acid Composition and Volatile Profile of *longissimus thoracis* *et* *lumborum* Muscle from Burguete and Jaca Navarra Foals Fattened with Different Finishing Diets

**DOI:** 10.3390/foods10122914

**Published:** 2021-11-24

**Authors:** Aurora Cittadini, Rubén Domínguez, Mirian Pateiro, María V. Sarriés, José M. Lorenzo

**Affiliations:** 1 Instituto de Innovación y Sostenibilidad en la Cadena Agroalimentaria (IS-FOOD), Universidad Pública de Navarra (UPNA), Arrosadia Campus, 31006 Pamplona, Spain; aurora.cittadini@unavarra.es; 2 Centro Tecnológico de la Carne de Galicia, Rúa Galicia No. 4, Parque Tecnológico de Galicia, San Cibrao das Viñas, 32900 Ourense, Spain; rubendominguez@ceteca.net (R.D.); jmlorenzo@ceteca.net (J.M.L.); 3 Área de Tecnología de los Alimentos, Facultad de Ciencias de Ourense, Universidad de Vigo, 32004 Ourense, Spain

**Keywords:** horsemeat, commercial feed, silage, organic diet, lipid profile, volatile compounds

## Abstract

The present study evaluated the effect of breed, Jaca Navarra (JN) *vs.* Burguete (BU), and finishing diet, conventional concentrate—diet 1 (D1) *vs.* silage and organic feed—diet 2 (D2), on the fatty acid composition and volatile profile of *longissimus thoracis et lumborum* muscle from forty-six foals. For this, foals were reared under a semi-extensive system and slaughtered at about 21 months of age. The outcomes showed that breed and finishing regime had a significant (*p* < 0.05) effect on the lipid and volatile profile of foal meat. In particular, JN foals reported higher polyunsaturated fatty acid contents and better nutritional indices in line with the health guidelines; whereas, BU and D1 groups generated higher amounts of total volatile compounds. However, it was the diet to occupy a central role in this study. Indeed, diet 2, due to its “ingredients” and composition, not only ameliorated the lipid profile of foal meat, but also reduced the generation of volatile compounds associated with lipid oxidation and minimized off-flavors. Thus, this diet could give an added value to the aromatic perception of meat and improve its sensorial acceptability.

## 1. Introduction

The consumption of horse meat, although still unpopular in many countries due to cultural and/or religious reasons [1], is slowly increasing. Foal meat, in fact, fits the modern market demands and could become an alternative red meat [2]. Nowadays, people’s attention is mainly focused on the quality of food, its beneficial effects and its “green image”. Indeed, foods with a healthy nutritional profile and which are environmentally-friendly are preferred by modern consumers [3]. In this sense, equines could be considered a “sustainable” source of high-quality meat [4,5,6,7]. They are non-ruminant domestic grazers and hindgut fermenters that can compete favorably with ruminants for the utilization of pastures and rangelands. In particular, owing to their unique digestive physiology, they are characterized by a higher intake capacity, lower methane emissions, and the ability to efficiently absorb and transfer dietary polyunsaturated fatty acids (PUFAs) (before the anaerobic microbial hydrogenation) from feed (pasture) to muscle tissues with very low deposition of trans-fatty acids [2,8,9]. Therefore, its fatty acid profile is usually described as “healthy” due to its high levels of essential and other PUFAs, such as α-linolenic (C18:3*n*-3) and other long-chain fatty acids, that have been reported to have beneficial properties for preventing chronic diseases [2,10,11]. In this regard, several studies have pointed out that horses produce nutritionally valuable meat, characterized by high-value proteins, iron, B type vitamins, as well as a low fat and cholesterol amounts and a favorable dietetic fatty acids profile [4,6,7,9,12,13]. On the whole, horse meat production is arousing interest due to its important role at social, economic and environmental levels: preservation the ecosystem of pasturages, protection the area against fire and erosion, maintenance of the population in rural areas, reduction of methane and other greenhouse gases, supply of food with enhanced *n*-3 fatty acid content, and the preservation and recovery of local breeds [2,5,8,14,15].

The “Jaca Navarra” (JN) and “Burguete” (BU) are endangered autochthonous breeds situated in Northern Navarre (Northeastern Spain) and have been included in the list of the Domestic Animal Diversity Information System hosted by Food and Agriculture Organization (FAO) [16]. Jaca Navarra is a light draught breed whose precise origin is unknown, while Burguete represents a medium-sized horse breed originated by the crossing of the local mares (Jaca Navarra) and foreign heavy stallion stock [17].

However, considering the increasing interest in horse meat nutritional properties, with special attention on its lipid composition, limited information is available about these two breeds. Concretely, some studies were conducted about Burguete foals [18,19,20], but scarce documentation is accessible about the quality and properties of Jaca Navarra foal meat. Additionally, aroma compounds released from meat are also very important for consumer satisfaction and acceptance. Meat aroma is complex since it is the product of a great variety and variability of compounds that provide a combination of many different flavor notes [21,22]. In this regard, the knowledge of these volatile compounds, responsible for aroma perception, is of great interest in order to obtain a high meat quality. As regards horse meat, a limited number of works were published about its volatile profile, mainly with application of thermal treatments and/or ageing times [23,24,25,26,27]. To date, the volatile compounds from BU and JN foals have still not been investigated. Moreover, the lipid fraction and especially the composition of fatty acids are considered one of the major contributors to the flavor development in meat [28,29].

In this context, it is worth highlighting that several studies have confirmed that breed and finishing diet, among others, can strongly affect horse meat composition and quality [7,9,18,30,31,32,33,34] and, as a consequence, also its fatty acid profile and flavor [29,35]. In particular, production system and feeding strategies employed play a decisive role on the quality of fresh horse meat [4,7,36]. Nevertheless, research is still insufficient and a deeper understanding of its nutritional characteristics and aromatic profile would be beneficial for the horse meat industry.

Therefore, the objective of the current work was to study the effect of breed (BU *vs.* JN) and finishing diet (conventional concentrates—diet 1 *vs.* silage and organic feed—diet 2) on the fatty acid composition and volatile profile of *longissimus thoracis et lumborum* muscle from forty-six foals.

## 2. Materials and Methods

### 2.1. Experimental Design and Animal Management

In this work, the trial involved forty-six foals, twenty-seven from Jaca Navarra (JN) and nineteen from Burguete breed (BU). A fuller description of the animal management and experimental design has been previously described in Cittadini et al. [3]. Briefly, foals were obtained from Navarre local farms after weaning and reared at pasture (on valley and mountain fields) until 17 months of age (for five months). At this point, the herd, at a mean age of 17 months of age, was finished indoors in the experimental farm of the Institute for Agri-food and Technology and Infrastructures of Navarre (Roncesvalles, Navarre, Spain) for 3–4 months. In particular, foals were submitted to two different dietary regimes, denominated diet 1 (D1) and diet 2 (D2). The D1 group included 22 foals (13 of JN and 9 of BU breeds), fattened with conventional concentrates (starter and finisher ones) and straw (*ad libitum*). Conversely, in D2 group, 24 foals (14 of JN and 10 of BU breeds) were supplemented with silage (produced by local farmers) and an organic fodder (with certification of UE/no UE Organic Agriculture), where silage formed the major part of the diet. The chemical composition and fatty acids profile (g/100 g of fatty acids) of feeds employed can be found in Table 1, while a fuller information on their ingredients was reported previously by Cittadini et al. [3]. Each group of foals followed an adaptation period (14 days) to finishing diets, where animals were gradually introduced to the commercial feeds using oats and silage, in order to avoid colics that normally occur with a sudden change in the diet. Hence, animals were separated into four groups: (JN-D1), (JN-D2), (BU-D1), and (BU-D2).

### 2.2. Animal Slaughter and Sample Collection

Foals, at a mean age of 21 months and with a live weight of about 466 kg [3], were transported to an accredited abattoir (Protectora de carne S.L., Salinas de Pamplona, Navarre, Spain) the day before slaughter, following the current EU regulations (Council Regulation 1/2005EC, 2005) [37], without mixing groups and trying to minimize the stress of the animals. The foals were stunned with a captive bolt, slaughtered and skinned, eviscerated, and washed according to the specifications outlined in the European legislation (Council Regulation 1099/2009, 2009) [38]. Immediately after the slaughtering procedures, hot carcass weight and dressing percentage were determined [3]. Successively, the carcasses were chilled for 24 h in a conventional room at 0 °C. At this point, the left half-carcasses were transported under refrigeration to the installations of Cárnicas Mutiloa (Sangüesa, Navarre, Spain) and stored in a chilling room at 0 °C for four days, according to the commercial Navarre procedure. Afterwards, the *longissimus thoracis et lumborum* (LTL) muscle was excised from each half-carcass (from the sixth to the thirteenth rib) under aseptic conditions. The LTL muscles were then cut into 2.5 cm thick steaks, vacuum packed and stored at −20 °C until subsequent analysis. In particular, in this work, determinations were carried out on the steak from the 7th rib. Prior to the analysis, each steak (about 80 g) was thawed overnight at 4 ± 1 °C, successively trimmed of external fat, connective tissue and tendons, and finally it was appropriately chopped and homogenized in order to obtain a representative sample of each animal.

### 2.3. Analysis of Fatty Acid Methyl Esters

For fatty acid determination, total fat was extracted according to the method described by Bligh and Dyer [39], with modifications. In brief, 10 g of sample was homogenized with NaCl (1%), in order to achieve a moisture of 80%, 8 mL of chloroform and 20 mL of methanol for 30 s (at 12,000 rpm) (Allegra^TM^ X-22 Centrifuge, Beckman Coulter, Brea, CA, USA). Then, 10 mL of chloroform was added to the mixture and homogenized, and after that, 10 mL of NaCl (1% in distilled water) was put in and mixed. Afterwards, the organic phase (lower layer containing chloroform and fat) was separated from the aqueous layer (upper phase) and residues (“meat phase”) by centrifugation at 4000 rpm for 10 min. Finally, the lower phase was transferred to a test tube (previously weighed) and evaporated to dryness using N_2_ on a TurboVap^®^ LV evaporator (1.2 bar nitrogen pressure with water bath temperature at 56 °C) (Zymark Corporation, Hopkinton, MA, USA). After cooling at room temperature, the test tube was weighed and the amount of fat was calculated by difference and reserved for transesterification. Fatty acid transesterification was carried out in accordance with the procedure formerly described by Barros et al. [40], with some modifications: 20 ± 5 mg of extracted fat was dissolved in 1 mL of toluene and successively mixed with 2 mL of sodium methoxide (0.5 N) solution, the mixture was allowed to stand for 15 min at room temperature after being vortexed for 10 s. Then, 4 mL of a H_2_SO_4_ solution (10% of H_2_SO_4_ in methanol) was added, vortexed for 10 s and allowed to stand for 5 min. At this point, 2 mL of saturated sodium bicarbonate solution was added and vortexed again. For the extraction of fatty acid methyl esters, 1 mL of hexane was added to the samples, vortexed for 10 s, and 970 µL of organic phase was then transferred to an appropriate vial for gas chromatography (GC) and mixed with 30 µL of C19:0 (10 mg/mL; nonadecanoic acid; internal standard).

Separation and quantification of fatty acids methyl esters (FAMEs) were performed using a gas chromatograph (GC-Agilent 7890B, Agilent Technologies, Santa Clara, CA, USA) equipped with a flame ionization detector (FID) and a PAL RTC-120 autosampler with a liquid injection tool (Pal System). One microliter of sample was injected in split mode (1:50). The injector was maintained at 250 °C and 64.2 mL/min of total flow. A DB-23 fused silica capillary column (60 m, 0.25 mm i.d., 0.25 µm film thickness; Agilent Technologies) was employed for FAMEs separation. Chromatographic conditions were as follows: initial oven temperature of 50 °C (held for 1 min), first ramp at 25 °C/min to 175 °C, second ramp at 3 °C/min to 230 °C (held for 3 min), and third ramp at 2 °C/min to a final temperature of 235 °C (held for 3 min). Helium was used as a carrier gas at a constant flow-rate of 1.2 mL/min, with the column head pressure set at 22.9 psi. The FID detector was maintained at 280 °C, while the operational flows were set as 40 mL/min of H_2_, 450 mL/min of air and 30 mL/min of makeup flow (He). The detector signal was recorded at 10 Hz data rate. The total time for chromatographic analysis was 32.83 min. Data acquisition and equipment control was carried out using the software Mass Hunter GC/MS Acquisition B.07.05.2479 (Agilent Technologies), while the data analysis was carried out with the software Mass Hunter Quantitative Analysis B.07.01. Individual FAMEs were identified by comparing their retention times with those of authenticated standards (FAME Mix-37 components; trans-11 vaccenic acid (11*t*-C18:1; TVA); cis-vaccenic acid (C18:1*n*-7, CVA, Supelco, Madrid, Spain); conjugated linoleic acid (9c,11*t*-C18:2, CLA, Matreya, State College, PA, USA) and docosapentaenoic acid (C22:5*n*-3; DPA)), and the results were expressed as g/100 g of fat.

Nutritional implications were assessed by calculating the amount of saturated fatty acids (SFA), monounsaturated fatty acids (MUFA), polyunsaturated fatty acids (PUFA), *n*-3, *n*-6 and long chain *n*-3 (LC *n*-3: eicosapentaenoic acid, C20:5*n*-3, EPA; docosapentaenoic acid, C22:5*n*-3, DPA; docosahexaenoic acid, C22:6*n*-3, DHA) fatty acids, as well as the *n*-6/*n*-3 and the PUFA/SFA ratios. Furthermore, the following parameters were calculated as follows: atherogenic (AI) [C12:0 + (4 × C14:0) + C16:0]/[(ΣMUFA) + (ΣPUFA)] and thrombogenic (TI) [C14:0 + C16:0 + C18:0]/[(0.5 × ΣMUFA) + (0.5 × *n*-6) + (3 × *n*-3) + (*n*-3/*n*-6)] indices [41], and hypocholesterolaemic/hypercholesterolaemic ratio (h/H) [Σ(C18:1*n*-9, C18:1*n*-7, C18:2*n*-6, C18:3*n*-3, C20:3*n*-6, C20:4*n*-6)/Σ(C14:0 + C16:0)] [42]. It is recognized that some fatty acids can help to prevent or promote coronary thrombosis and atherosclerosis based on their effect on low-density lipoprotein (LDL) concentration and serum cholesterol. In particular, AI and TI indices indicate the effects of fatty acids on cardiovascular risk, while the h/H ratio reflects the functional effects of fatty acids on cholesterol metabolism [41,42].

### 2.4. Volatile Compound Analysis

The extraction, separation, identification and determination of volatile compounds were carried out following the procedure and conditions first described by Domínguez et al. [22]. Shortly, solid-phase microextraction (SPME) with an autosampler Pal RTC-120 was employed for the separation of volatile compounds, while a gas chromatograph 7890B GC-System (Agilent Technologies, Santa Clara, CA, USA) coupled to a mass selective detector 5977B MSD (Agilent Technologies) was used for their separation, identification and quantification.

In particular, the SPME tool was loaded with a fused-silica fiber (10 mm length) coated with a 50/30 mm thickness of DVB/CAR/PDMS (divinylbenzene/carboxen/polydimethylsiloxane) (Supelco, Bellefonte, PA, USA). Prior to analysis, fiber was conditioned by heating in a SPME Fiber Conditioning Station at 270 °C for 30 min. Meanwhile, 1 ± 0.02 g of minced meat was weighed in a 20 mL vial (Agilent Technologies, Santa Clara, CA, USA) and immediately screw-capped with a laminated Teflon-rubber disc, for headspace SPME (HS-SPME) extraction of volatile compounds. Samples were equilibrated for 15 min at 37 °C, and successively the extraction process was carried out for 30 min at the same temperature (ensuring a homogeneous temperature for sample and headspace).

After extraction, the fiber was desorbed and maintained at 260 °C during 8 min in the injection port of the gas chromatograph–mass spectrometer (GC-MS) system (splitless mode; helium pressure 9.59 psi). Helium was used as the carrier gas with a constant flow of 1.2 mL/min. For volatile separation, a DB-624 capillary column (30 m, 0.25 mm i.d., 1.4 µm film thickness; J&W Scientific, Folsom, CA USA) was employed. The oven temperature program was isothermal for 10 min at 40 °C, raised to 200 °C at a rate of 5 °C/min, successively raised/increased to 250 °C at a rate of 20 °C/min, and held for 5 min (total run time for analysis was 49.5 min). Injector and detector temperatures were both set and maintained at 260 °C. The mass spectra were acquired using the 5977B selective detector working in electronic energy at 70 eV, with an electron multiplier voltage of about 900 V and collecting data at 2.9 scans/s over the range *m/z* 40–550 in scan acquisition mode. The mass source was maintained at 230 °C, while the mass quad was set at 150 °C.

After chromatographic analysis, data processing and identification were realized with the software MassHunter Quantitative Analysis B.07.01 (Agilent Technologies), where the integration was carried out with the Agile2 algorithm, while peak detection was realized by deconvolution. Compounds identification was accomplished by comparing their mass spectra with those included in the NIST14 library (National Institute of Standards and Technology, Gaithersburg, MD, USA) (2014, version 2.2). In particular, the volatile compounds were considered appropriately identified when their spectra exhibited a library match factor > 85%, and were included in the data analysis only if they appeared in more than 50% of the group of samples (*e.g.*, in 10 out of the 19 and 14 out of the 27 samples of foal meat from the BU and JN breeds, respectively). Finally, after integration, peak detection and identification of each compound, the extracted ion chromatogram (EIC) from the quantifier ion was obtained from each peak. The final outcomes were expressed as area units of the EIC × 10^4^ per gram of fresh sample (AU × 10^4^/g of fresh meat).

### 2.5. Statistical Analysis

Statistical analyses were performed using the SPSS statistical software (SPSS 25.0, Chicago, IL, USA). After a prior verification of normal distribution (Shapiro–Wilk test) and variance homogeneity (Levene test), the effect of breed, finishing diet and the interaction between breed × finishing diet on fatty acids and volatile compound content was exanimated using analysis of variance (ANOVA) with the general linear model (GLM) procedure, where these parameters were set as dependent variables, and breed and finishing diet as fixed effect. In all analyses, the least squares mean was compared using Duncan’s *t*-test. Significance was indicated at *p* < 0.05.

## 3. Results and Discussion

### 3.1. Effect of Breed and Finishing Diet on Fatty Acids Profile

The fatty acids contents (g/100 g of fat) and health indices of foal meat are shown in Table 2 (only those represented > 0.05%). In this study, the foal meat fatty acids are predominated by MUFA, followed by SFA and finally PUFA for all groups concerned (MUFA < SFA < PUFA). This trend is consistent with data previously reported by other authors [7,33,34,43,44], studying the lipid profile of meat from foals of different breeds and reared under a semi-extensive system. However, other studies showed SFAs as predominant in horse meat finished with concentrate [7,9,13,18,20,30,31,45], or PUFAs in animals managed under extensive livestock systems [9,14,34].

In particular, our data reported that breed significantly affected the fatty acids profile (*p* < 0.05) of both groups. In line with literature, different works found “breed effects” on lipid profile of foals from different breeds finished with the same diet [18,30]. Although both BU and JN foals showed that MUFAs were the most abundant fraction, followed by SFAs and PUFAs, significant differences were detected between the two groups. As regards SFA, JN foals showed a greater amount compared to BU ones (33.41 *vs.* 32.23 g/100 g of fat) (*p* < 0.05). This could be explained especially by their higher contents of palmitic (C16:0) (though not significant) and stearic (C18:0) (*p* < 0.001) acids. Total SFA contents recorded in both breeds are in accordance with values reported in the scientific literature for horse meat (34–49% of total FA) [2,4,46]. In detail, considering the majority fatty acid, C16:0, both groups presented similar amounts and corresponding to those found in 24-month female BU foals [20] and in 11-month male Italian Heavy Draught Horses (IHDH) [13]. Conversely, JN foals showed C18:0 values comparable to the contents obtained by Lorenzo et al. [9] and Beldarrain et al. [10] in Galician Mountain (GM) and Hispano-Bretón (HB) horse breeds, respectively. On the contrary, the BU group reported C18:0 contents less than those previously reported by other authors [18,20], who studied the same foal breed. This dissimilarity could be probably related to the different age, diet and duration of the finishing diets. On the other hand, statistical analysis indicated that the BU group recorded higher MUFA values compared to the JN group (37.35 and 35.58 g/100 g of fat), although without significant differences. Among MUFAs, in fact, oleic (C18:1*n*-9) acid represented the most abundant and showed similar values in both groups (*p* > 0.05). In this case, our outcomes resulted to be higher than those described in recent works [7,10,44,45]. Considering PUFAs, the JN group reported higher content than the BU one (*p* < 0.001) (15.68 *vs.* 12.85 g/100 g of fat). Although our results were lower than those reported by other authors [9,13,18,20,30,31,43,44], they could be considered within the range of values described by Lorenzo et al. [46] (16–46% of total FA). Among *n*-3 PUFAs, α-linolenic acid (C18:3*n*-3) resulted to be most abundant in JN than in BU foals, though not significantly (*p* > 0.05). Similar values were previously found in IHDH foals by some researchers [13,31]. At the same time, our results for BU foals followed a different trend compared to those showed in studies about the same breed [18,20]. Whereas, statistical differences were detected in long chain (LC) *n*-3 PUFAs, where the BU group showed lower contents compared to the JN group (*p* < 0.05), probably due to its minor DPA value (*p* < 0.01). Nevertheless, statistical analysis did not indicate differences in *n*-3 PUFA content between the two groups. On the other hand, *n*-6 PUFAs were significantly affected by breed (*p* < 0.001), especially linoleic (C18:2*n*-6) and arachidonic (C20:4*n*-6) acids. In fact, these fatty acids showed significant higher concentrations in JN samples in comparison to BU ones (*p* < 0.001). However, our results showed lower values than those reported in literature for C18:2*n*-6 [2,7,44] and for C20:4*n*-6 [7,20,30]. Concerning the nutritional indices, *n*-6/*n*-3 and PUFA/SFA ratios also resulted to be affected by breed (*p* < 0.01). As described above, breed had a significant effect on *n*-6 PUFA contents, affecting, as a consequence, the *n*-6/*n*-3 ratio, which recorded the lowest values in BU foals compared to JN ones (2.24 *vs.* 2.62, respectively). Nevertheless, both BU and JN samples obtained *n*-6/*n*-3 values lower than 4, according to the recommendations [47]. These results could be considered more favorable in comparison with those obtained in previous studies [13,18,20,30,31] (3.70–15.56). In contrast, other authors obtained lower values than ours from GM, GM × HB or GM × BU foals [7,9,33,43]. However, it is worth mentioning that outcomes derived by the *n*-6/*n*-3 ratio should not be considered alone. Concerning the PUFA/SFA ratio, in line with aforementioned data, JN had a greater ratio than BU (0.47 *vs.* 0.40, respectively), complying with recommendations for this ratio (>0.4) [48]. Nonetheless, these outcomes are slightly below those found in literature [46] (0.48–1.15). On the other hand, statistical analysis indicated that TI, AI and h/H indices were unaffected by breed, with results reporting similar values in both breeds (a mean of 0.88, 0.74 and 1.51, respectively). According to the recommendations, TI and AI indices should be as low as possible [41], while h/H should be high [42]. Our outcomes are in agreement with the range of values observed in previous studies [7,20,34,45,49], 0.47–1.39 for TI, 0.50–1.00 for AI, and 1.09–2.26 for h/H. Thus, considering our outcomes, JN groups suggested to produce a meat with a healthy fatty acids profile. The distinct fatty acid distribution in intramuscular fat from BU and JN foals could be related to various factors. Among them, a hypothesis could be that these breeds have different endogenous enzymatic activities (desaturase and elongase enzymes) [48]. Another possible explanation could be related with the de novo synthesis of fatty acids, which are correlated with the fat deposition. This phenomenon determines that the high fat deposition can be due to a higher de novo synthesis, which is characterized by the synthesis of both SFA or MUFA, while samples with low fat contents imply higher direct deposition of fatty acids from the diet (in this case, PUFAs). This fact agrees with our results and explains the differences between both foal breeds, since BU presenting higher MUFA also had the highest amount of fat (5.01%; data not shown), while JN with higher PUFAs content had the lowest amounts of fat (3.79%; data not shown).

In addition, the lipid profile displayed to be highly affected by the type of finishing diet. Literature confirmed that the dietary treatments could be responsible for significant changes in the fatty acid composition of foal meat [7,9,33,34,43]. As aforementioned, the intramuscular fat of animals from D1 and D2 groups presented MUFAs as the most abundant fraction, followed by SFAs and then PUFAs. Nevertheless, significant differences were found between the two dietary treatments. The D1 group reported the highest (*p* < 0.01) values of SFAs compared to the D2 group (33.89 *vs.* 32.04 g/100 g of fat, respectively), mainly influenced by palmitic (C16:0) (*p* < 0.05), stearic (C18:0) (*p* < 0.01) and miristic (C14:0) (*p* < 0.001) acids, resulting to be the most plentiful SFAs in this group, in descending order (C16:0 < C18:0 < C14:0). This trend is consistent with data published in literature [2], where C16:0 resulted to be the predominant fatty acid. Moreover, our SFA amounts are in accordance with values reported in previous studies about horse meat [46]. Considering MUFA contents, also in this case, D1 foals presented greater (*p* < 0.001) values than D2 ones (39.06 *v*s. 33.80 g/100 g of fat, respectively), probably due to their higher (*p* < 0.001) C18:1*n*-9 contents. These values disagree with data showed in literature [2], since our outcomes are above those usually reported in most of studies in horses managed under semi-extensive systems. On the other hand, the “predominance” of D1 groups reflected the fatty acids profile of feeds administered to animals (Table 1). In fact, silage, the main component of D2, reported the lowest content of MUFA, about tenfold less than those in D1. On the other hand, D2 foals recorded higher (*p* < 0.01) PUFA values compared to the D1 group (15.51 *vs.* 13.41 g/100 g of fat, respectively). In the same manner, in fact, the D2 group showed significantly greater (*p* < 0.001) *n*-3 PUFA contents than the D1 group (5.25 *vs.* 3.43 g/100 g of fat, respectively). These outcomes could be justified mostly by the higher (*p* < 0.05) amounts of C18:3*n*-3 and LC *n*-3 recorded in the D2 group in comparison to the D1 one. These data are comparable to the values obtained in scientific literature [2,4], including studies about semi-extensively reared foals. On the contrary, our results were lower than those obtained from foals managed under extensive conditions [9,49,50], characterized by a considerable deposition of *n*-3 PUFAs. According to studies, C18:3*n*-3 is generally found in grass, accounting for between 50–60% of the total fatty acid profile, and its content in meat can be directly correlated to the dietary (pasture and silage) intake of the animal [20]. Moreover, Sahaka et al. [51] stated that a specific pancreatic enzyme (pancreatic lipase related protein 2), able to hydrolyze the C18:3*n*-3 esterified in plant galactolipids, is exclusively present in horses, allowing the deposition of this fatty acid in equine tissues. Recent studies pointed out that while SFAs and MUFAs result mainly from de novo synthesis, PUFAs result principally from the diet [52]. Therefore, and taking into account a previous comment, considering the diets of our groups, the high level of C18:3*n*-3 in the D2 group could be expected. In fact, silage contained the greatest amounts (53.71% of total FA) of this valuable fatty acid, as shown in Table 1. Thus, coinciding with the literature [2,10], our outcomes confirmed that diet could be considered the main source of variation in the fatty acid composition of foal meat, and C18:3*n*-3 is principally responsible for higher *n*-3 contents. On the other hand, similar *n*-6 levels were found among groups (*p* > 0.05). Overall, these results may be expected, considering the composition and lipid profile of diets showed in Table 1, where D1 recorded higher fat percentage, SFA and MUFA contents and lower PUFA amounts compared with the “ingredients” of D2. As a consequence, diet had also a significant impact on nutritional indices, where D2 reported better values than the D1 group. In particular, the D2 group presented a more favorable (*p* < 0.001) *n*-6/*n*-3 ratio than D1 (2.00 *vs.* 2.97, respectively), albeit both treatments complied with the recommended values for human health, recording values less than 4 [47]. Furthermore, our outcomes reported lower values than those observed in most of the recent studies about foal meat [2,10,44,45]; whereas, D2 reported significantly greater (*p* < 0.001) PUFA/SFA values than D1 (0.49 *vs.* 0.40, respectively), and in line with the recommendations (>0.4) [48]. Other authors [18,20,43] published outcomes higher than ours. However, our data can be considered better than those recorded for beef and sheep meat [46]. Moreover, D2 foals recorded the lowest (*p* < 0.001) TI (0.83 *vs.* 0.94, respectively) and AI (0.72 *vs.* 0.76, respectively) (*p* < 0.01) values and the highest h/H ratios (1.54 *vs.* 1.48, respectively) (even not significantly), according to the recommendations [41,42]. Sarriés et al. [20] observed higher TI values than our levels, but a similar AI value in female BU foals slaughtered at 24 months of age. As regards the h/H ratio, our results were higher than those described in Catria horses [45]. As a consequence, the results suggest that D2 improved the fatty acid profile and nutritional value of foal meat, confirming that the type of finishing diet has the potential to affect both FA composition and the nutritional properties of meat.

Considering the four groups, in line with our outcomes, JN-D2 was suggested to be the group with a promising fatty acid and nutritional profile. Nonetheless, no significant interactions between the main categories (breed × finishing diet) were found (*p* > 0.05), except for cis-vaccenic acid (C18:1*n*-7).

### 3.2. Effect of Breed and Finishing Diet on Volatile Compounds

The effect of breed and finishing diet on the volatile compounds in the headspace of raw foal steaks (expressed as AU × 10^4^/g fresh meat) is presented in Table 3 and Table 4. Concretely, a total of 62 volatile substances were isolated and identified using the SPME/GC-MS technique. Although the SPME technique is not normally employed for absolute quantifications, when the same extraction methodology is used, this method allows the determination of the relative amounts between samples [26,53]. The compounds obtained were divided into ten families according to their chemical nature: thirteen hydrocarbons (lineal, branched and cyclic), three acids, fourteen alcohols, nine aldehydes, six ketones, six esters, four ethers, two furans, four nitrogen compounds, and one sulfur compound.

#### 3.2.1. Hydrocarbons: Lineal, Branched and Cyclic

Table 3 shows the influence of breed and finishing diet on hydrocarbon compounds of *longissimus thoracis et lumborum* muscle of foals. The thirteen substances pertaining to this volatile family were detected in all treatments and were distributed as follows: five lineal hydrocarbons, three branched hydrocarbons, and five cyclic hydrocarbons.

As can be seen, breed significantly (*p* < 0.01) affects the total hydrocarbon content, being higher in BU foals compared with JN foals (2919 *vs.* 2462 AU × 10^4^/g fresh meat, respectively). Similarly, the BU group also presented (though not significantly) the highest concentrations of the total lineal (3.97 *vs.* 3.92 AU × 10^4^/g fresh meat, respectively), branched (*p* < 0.001; 4.12 *vs.* 2.96 AU × 10^4^/g fresh meat, respectively) and cyclic (*p* < 0.01; 2911 *vs.* 2455 AU × 10^4^/g fresh meat, respectively) hydrocarbons. Nevertheless, our results were lower than those reported by other authors, who studied the volatile profile of raw meat from GM × HB [25,27] and GM [26] foals. However, it is worth highlighting that in these studies [25,26,27], although SPME methodology was employed, a different data processing methodology (total ion chromatogram—TIC) was used, while in our study, the extracted ion chromatogram (EIC) is considered for peak detection and quantification. Therefore, our results are not exactly comparable with these works.

**Figure 1:**
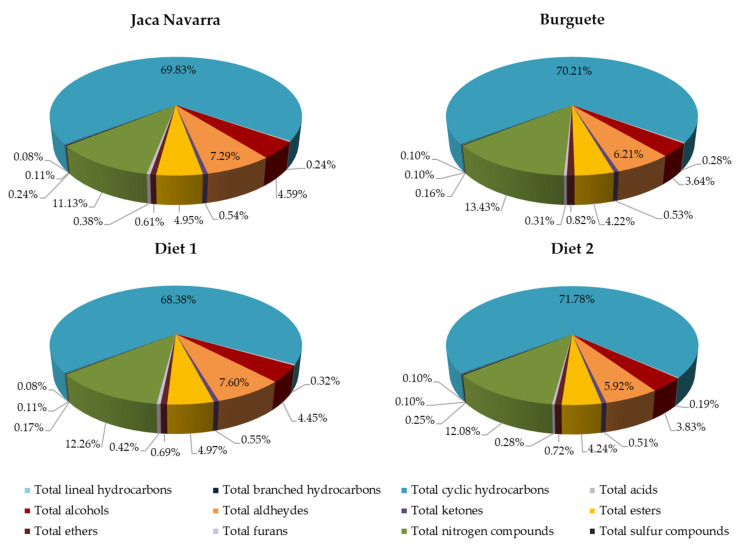
Volatile families of *longissimus thoracis et lumborum* muscle from foals (displayed as percentage) influenced by the breed and finishing diet.

Regarding the individual hydrocarbons, most of these volatile compounds were significantly (*p* < 0.05) influenced by the breed. Among linear hydrocarbons, the most abundant compound, octane, presented similar amounts in both breeds (*p* > 0.05). However, significant differences were reported in minority compounds, such as nonane, higher (*p* < 0.001) in BU foals than in JN ones (0.76 *vs.* 0.54 AU × 10^4^/g fresh meat, respectively), and dodecane, recording the highest (*p* < 0.001) content in JN foals (0.31 *vs.* 0.14 AU × 10^4^/g fresh meat, respectively). Regarding branched hydrocarbons, the majority compound, pentane, 3-methyl, reported significantly greater (*p* < 0.001) values in BU foals compared to JN ones (3.48 *vs.* 1.81 AU × 10^4^/g fresh meat, respectively); whereas, bicyclo[3.2.0]hepta-2,6-diene represented not only the most abundant cyclic hydrocarbon, but also the majority compound within the entire hydrocarbon family. BU foals, also in this case, displayed the highest (*p* < 0.01) values of bicyclo[3.2.0]hepta-2,6-diene compared to the JN group (2887 *vs.* 2432 AU × 10^4^/g fresh meat, respectively). Therefore, considering the contribution of hydrocarbons over the total volatile compounds, this group represented the first most plentiful family in both breeds, as a total percentage of 70.41% in BU foals and 70.03% in the JN group was obtained (Figure 1). This trend disagrees with results obtained by other researchers, who found that esters represented the most abundant volatile compounds in raw steaks from GM × HB [25,27] and GM [26] horses.

In addition, statistical analysis showed that the total content of hydrocarbons was significantly affected also by the type of finishing diet (*p* < 0.05). In fact, the D1 group reported the highest values compared with D2 foals (2832 *vs.* 2484 × 10^4^/g fresh meat, respectively) (data not shown). Moreover, D1 animals produced significantly higher amounts of total lineal (*p* < 0.001; 4.45 *vs.* 3.48 AU × 10^4^/g fresh meat, respectively) and cyclic hydrocarbons (*p* < 0.05; 2824 *vs.* 2477 AU × 10^4^/g fresh meat, respectively); whereas, both groups reported similar values of total branched hydrocarbons (*p* > 0.05). These results are in contrast with those showed in literature for foals finished with concentrates [25,27], recording higher values compared to ours. On the other hand, the outcomes for the D1 group are in agreement with those reported by other authors [54], who observed the highest total hydrocarbon contents in animals fattened with conventional concentrate.

Considering the singular trends, most individual hydrocarbons were also affected by the finishing diet, apart from pentane, 2-methyl, cyclopentane-methyl and dodecane. Besides, D2 foals generally displayed lower amounts of hydrocarbons than the D1 group, except for undecane, 5,5-dimethyl- (*p* < 0.001). Moreover, in both finishing diets, the lineal, branched and cyclic hydrocarbons with the highest amounts were the same, specifically nonane, pentane, 3-methyl and bicyclo[3.2.0]hepta-2,6-diene, respectively. However, the D2 group showed values of 1.55, 2.20 and 2457 AU × 10^4^/g fresh meat for these singular compounds (respectively), which were significantly lower (*p* < 0.05) than those from the D1 group, with 2.04, 2.83 and 2797 AU × 10^4^/g fresh meat. Thus, D1 foals tended to report the greatest amounts in most of hydrocarbons, probably related to the high content of C18:1*n*-9 (Table 2). In fact, Dominguez et al. [29] reported that degradation of this fatty acid generates hydrocarbons, among them octane and nonane. According to the finishing diet, also in this case, this family of volatile compounds represented the major percentage over the total volatile substances (Figure 1).

Considering the four treatments, it is evident that BU-D1 had the highest values of total hydrocarbons (3015 AU × 10^4^/g fresh meat), followed by BU-D2 (2832 AU × 10^4^/g fresh meat), JN-D1 (2705 AU × 10^4^/g fresh meat) and JN-D2 (2235 AU × 10^4^/g fresh meat). Among the hydrocarbons, BU-D1 also showed the greatest amounts of octane and nonane, probably related to the high content of C18:1*n*-9 [29]. On the other hand, no significant interactions were detected among the main categories (breed × finishing diet), except for cyclopentane, 1,1-dimethyl-, cyclopropane and undecane, 5,5-dimethyl-.

However, although lineal, branched and cyclic hydrocarbons were found in a higher proportion in our samples, they have no relevance in the volatile pattern of meat, since previous studies showed that their contribution to the aroma of meat is minimal due to its high odor threshold value [29,55].

#### 3.2.2. Acids

In this study, three distinct acids were identified in our samples (Table 3). As can be seen, the breed significantly (*p* < 0.05) affected both the total amounts of acids and each individual compound values. In particular, BU foals reported significantly greater (*p* < 0.001) total content than JN foals (11.72 *vs.* 8.30 AU × 10^4^/g fresh meat). Unsurprisingly, this trend is also reflected in the singular acids identified, where the BU group showed significantly higher (*p* < 0.05) amounts, apart from butanoic acid, 4-hydroxy-. This latter compound, in fact, represented the most abundant acid identified in JN foals (*p* < 0.05), while the hexanoic acid is the predominant in BU group (*p* < 0.001).

These compounds were not detected in raw meat from GM × HB foals [25,27], so they could be considered characteristic of these autochthonous Navarre foal breeds. However, deeper investigations are necessary to define these volatiles compounds as potential breed biomarkers. The contribution of acids on the total volatile compounds was very low. Indeed, this family showed to be the second with the least presence in BU foals and the least plentiful in JN group. More concretely, total acids represented 0.28 and 0.24% in foals from the BU and JN breeds, respectively (Figure 1).

Furthermore, statistical analysis revealed that acid compounds (both total and singular compounds) were strongly (*p* < 0.05) influenced by the type of finishing diet (Table 3). Actually, D1 foals showed a total content significantly (*p* < 0.001) higher than D2 ones, and individual acids followed the same trend. The D2 group displayed the lowest (*p* < 0.001) amounts of the singular compounds detected in comparison with D1 foals. In particular, hexanoic acid showed to be the most abundant compound in the D1 group, representing 54.88% of the total acids. This outcome could be justified by the fact that D1 foals also reported the highest concentrations of C18:1*n*-9 (Table 2). A recent investigation actually stated that hexanoic acid could be originated by lipid degradation, and especially by this fatty acid [29]. Considering the total volatile compounds, also in this case, this class of family represented a low percentage. In fact, D1 and D2 generated only 0.32 and 0.19% of total acids, respectively.

Therefore, statistical analysis showed significant differences among the four groups, where the BU-D1 group generated the major amounts of acids (15.48 AU × 10^4^/g fresh meat), while JN-D2 foals reported the lowest values (5.20 AU × 10^4^/g fresh meat). However, significant interactions among the main categories (breed × finishing diet) were not found (*p* > 0.05).

#### 3.2.3. Alcohols

Table 3 shows the effect of breed and finishing diet on alcohols identified in the *longissimus thoracis et lumborum muscle* of foals. Fourteen compounds belonging to this family were detected both in JN and BU foals and their total content resulted to be unaffected by breed (*p* > 0.05). On the other hand, six of the alcohols detected showed significant differences among groups (*p* < 0.05). This could be explained by the fact that some alcohols have metabolic origin [56], as a consequence, genetic factors such as breed can influence their synthesis. In addition, other alcohols can also derive from lipid oxidation, since they are associated to the degradation of their homologous aldehydes [22,29]. Among them, 1-hexanol, showing higher (*p* < 0.01) values in BU foals, is associated to the reduction of hexanal [57] and the oxidation of the oleic acid [29]. Indeed, as can be seen in Table 2, the BU group reported greater concentrations of this fatty acid, though not significantly (*p* > 0.05). On the other hand, 1-octen-3-ol, being more abundant (*p* < 0.05) in JN foals, is considered to be a product of the autoxidation of linoleic and other polyunsaturated fatty acids [22] and it is distinguished by a mushroom-like grassy odor [58,59]. BU foals, in fact, showed significantly (*p* < 0.001) lower values of C18:2*n*-6 in comparison with JN ones (Table 2). These compounds were also identified in GM × HB foals [27], but their total contents were much greater than ours. Regarding the contribution of alcohols on the volatile content, this family was the fifth most plentiful, representing 3.64 and 4.59% of the volatile profile for BU and JN foals, respectively.

On the other hand, the type of finishing diet remarkably influenced the total alcohol content, where the D1 group reported the highest (*p* < 0.001) values. The same tendency was observed in each compound belonging to this family, reporting significant (*p* < 0.05) differences among groups, except for three alcohols (cyclobutanemethanol, prenol and cis-2-pentenol). In particular, the alcohol found in the highest quantity was 1-octen-3-ol for both foal groups (91.33 *vs.* 70.35 AU × 10^4^/g fresh meat for D1 and D2 foals, respectively), followed by 1-pentanol (41.55 *vs.* 28.03 AU × 10^4^/g fresh meat for D1 and D2 foals, respectively). Our outcomes are consistent with those published by some authors, who observed higher contents of these alcohols in animals fed with concentrates [54]. In particular, these compounds are described as products of lipid oxidation, especially of linoleic acid [22,29], typically found in high amounts in concentrates. Table 1, in fact, showed that concentrates employed in both diets reported relevant amounts of this fatty acid; however, considering the lipid profile of D1 and D2 foals (Table 2), similar values were recorded in both groups (*p* > 0.05). Furthermore, 1-hexanol, 1-heptanol and 1-octanol are described as typical volatile compounds of oxidizing oleic acid [29]. These alcohols were generated in higher (*p* < 0.001) amounts by D1 foals, which in fact also showed the greatest values of C18:1*n*-9 (Table 2). Corresponding with us, other authors observed the presence of 1-hexanol [27,60], 1-heptanol [60] and 1-octen-3-ol in raw meat from foals and heifers fed with concentrates. Nevertheless, our values were lower than those detected by Lorenzo et al. [27]. Moreover, among these alcohols, 1-octen-3-ol odor, as commented above, is described as “fungus” [59], but other compounds play a major role in the final aroma of meat. This is the case mainly of 1-pentanol, characterized by its pleasant, sweet or fruity odor, and 1-hexanol, which has a positive aroma described as herbal and with fatty notes [22,58,61]. Despite this, alcohols are characterized by a high odor threshold and their contribution to volatile flavor is minor compared to other volatile substances, such as aldehydes [22,62]. Regarding the involvement of alcohols on the total volatile content, this family was the fifth most abundant in both diet regimes, since it represented 4.45 and 3.83% of the volatile profile for D1 and D2 foals, respectively (Figure 1). Thus, these compounds probably had a low impact on the aroma of meat, considering also their usually high detection thresholds [62].

The effect of diet was particularly observed in JN foals, where JN-D1 presented the highest alcohol total content, while its counterpart, JN-D2, showed the lowest. In addition, significant interactions B × D were detected in the total contents of this family, except for glycidol, 1-hexanol, 1-heptanol, diethylene glycol, 2-ethyl-1-hexanol, 1-octanol and 6-undecanol.

#### 3.2.4. Aldehydes

Table 4 shows the effect of breed and finishing diet on the nine different aldehydes and their total contents detected in the *longissimus thoracis et lumborum* muscle. As showed in the table, the total content of these volatile compounds as well as most of individual aldehydes (6 of the 9 substances identified) were not affected by breed (*p* > 0.05), presenting similar values in both groups. On the other hand, propanal, 2-propynal and heptanal showed higher (*p* < 0.05) amounts in BU foals than JN ones. Researchers [22,29] affirmed that propanal and heptanal derived from oxidizing linolenic and oleic acids, respectively. However, our results contrast with the similar content of these fatty acids found in meat from JN and BU foals (Table 2). Moreover, hexanal was the most plentiful in the two groups but it did not present significant (*p* > 0.05) differences among them. Its predominance over the other compounds of this family can be related to the multiplicity of its synthesis pathways. In fact, some authors [29,63] asserted that this compound can be generated both from oleic, linoleic and arachidonic acids, and through the deterioration of other unsaturated aldehydes, such as 2,4-decadienal. Considering the lipid profile of our samples (Table 2), although both breeds showed significantly different (*p* < 0.05) values of C18:2*n*-6 and C20:4*n*-6, C18:1*n*-9, the predominant fatty acid among groups, presented similar values (*p* > 0.05). Thus, our outcomes could be justified by the behavior of this more abundant fatty acid. With regards to the contribution of aldehydes over the total volatile content, this is the third most abundant family for both groups (Figure 1). Recent studies [25,26] about raw foal meat agree with our outcomes, reporting this family as the third most plentiful in relation to the total volatile compounds detected. However, these studies showed greater total aldehyde content than ours. Lastly, it is worth highlighting that this class of compounds represents one of the main groups of volatile compounds owing to its low odor threshold, which can be critical for the aromatic profile of meat [64].

On the other hand, data showed that finishing diet significantly (*p* < 0.001) affected total aldehyde amounts, being higher in the D1 group in comparison with D2 foals (314.05 *vs.* 204.31 AU × 10^4^/g fresh meat, respectively). These data are consistent with those found by other authors [54,65,66], who detected higher amounts of these compounds in animals fattened with concentrates. Some researchers [65] related these results to the high level of linoleic acid usually present in the concentrates and the protective action of antioxidants (as carotenoids and tocopherols), abundant in forages, on the oxidation of fat from grass-fed animals [29]. Indeed, it was observed that concentrate-fed animals are more disposed to lipid oxidation than grass-fed animals, and this effect is due to the fact that forages are a natural source of antioxidants, presenting increased levels of vitamins A, C, and E, flavonoids, and carotenoids [28]. In fact, although grass-based diets and grass-fed animals are normally characterized by greater amounts of PUFA and, as a consequence, could be associated to higher lipid degradation [29], the antioxidants naturally present in forages reduce lipid oxidation rates and favor meat lipid stability [67,68]. In this sense, our outcomes can be justified by the fact that silage, mainly constituting the D2 regime, probably present antioxidant compounds able to prevent meat lipid oxidation. Recent studies, in fact, demonstrated that conserved forage, as silage, is still a significant source of antioxidants [69,70]. Moreover, the organic feed, used in diet 2, contained higher quantities of vitamin A (10,000 UI/kg) and E (15 mg/kg) in comparison with the conventional concentrates employed for the diet 1 (4800 UI/kg of vitamin A and 12 mg/kg of vitamin E) [3]. Thus, D2 seems to be a promising combination able to limit meat lipid oxidation and as a consequence, the generation of aldehydes. In the same way, the predominance of the D1 group on the D2 one was also observed in most of the individual aldehydes, apart from propanal, 2-propynal and pentanal. In particular, the main and predominant aldehyde in both diets was hexanal, representing one of the best indicators of lipid oxidation as previously commented [22,29]. This compound occupied the highest percentages within this family, and D1 foals presented significantly higher values than the D2 group (82.30 *vs.* 80.46%, respectively). Similarly, this tendency was observed also for other relevant aldehydes, such as heptanal, octanal and nonanal, described by Domínguez et al. [29] as characteristic volatile compounds of oxidizing oleic acid. These aldehydes in fact reported the highest (*p* < 0.001) values in D1 foals, which in turn presented also the greatest (*p* < 0.001) concentrations of this fatty acid (Table 2). Finally, also in this case, this family of compounds represented the third most plentiful in relation with the total volatile compounds of both groups. Thus, considering the low odor threshold of aldehydes, these volatile substances could have a relevant influence on the aromatic profile of our samples. According to the data published by some authors [22], hexanal can give rancid aroma at high levels, while in low quantities it has a pleasant grassy aroma. In addition, heptanal, octanal and nonanal have been related to pleasant meaty notes; octanal contributes meat-like, green, fresh, grassy and fruity notes, whereas nonanal imparts sweet and fruity aromas [22]. As previously commented, our values are lower than those observed by other authors [54,65,66]; consequently, from our samples, a pleasant grassy aroma could probably be detected. Nevertheless, it is complicated to associate an aldehydic compound to an animal feeding regime and be recognized as a source of a specific aroma in our samples.

As regards to the four groups of treatment, the highest amounts of aldehydes were generated from JN-D1 (349.6 AU × 10^4^/g fresh meat), followed by BU-D1 (262.6 AU × 10^4^/g fresh meat), BU-D2 (252.4 AU × 10^4^/g fresh meat) and JN-D2 (169.9 AU × 10^4^/g fresh meat). On the whole, total contents and most of singular aldehydes were affected (*p* < 0.05) by the interaction of breed and type of diet, except for 2-propynal, heptanal, octanal and 2-octenal, (E)-.

#### 3.2.5. Ketones

As shown in Table 4, six ketones were detected. As regards the breed effect, significant differences were observed among BU and JN foals. Concretely, total ketones were shown to be significantly the most abundant (*p* < 0.01) in the BU group compared with the JN one (21.86 *vs.* 18.90 AU × 10^4^/g fresh meat, respectively). These dissimilarities could be related to the fact that some ketones, including certain lactones, have a metabolic origin [56], and that their synthesis can be influenced by breed [71]. Moreover, our outcomes were shown to be largely lower than the amounts detected in GM × HB and GM foals [25,26,27]. On the other hand, considering the individual ketones, out of six volatile compounds identified, only two substances (2,3-pentanedione and butyrolactone) were significantly affected by breed (*p* < 0.05). Among them, 2,3-pentanedione represented the predominant ketone in BU foals, showing the highest amounts (*p* < 0.001), whereas butyrolactone showed greater (*p* < 0.05) amounts in JN foals. The presence of higher quantities of butyrolactone in JN could be justified by its significantly higher levels of C18:*2n*-6 (Table 2), since recent studies found that the lipid oxidation of this fatty acid can generate this type of lactone [64]. On the whole, this family of compounds corresponded to small percentages in relation to the total volatile compounds content, being the seventh most abundant group for both breeds (Figure 1).

As can be seen, the type of finishing diet enormously affected both the total ketones content and the individual amounts, except for 2,3-pentanedione. Indeed, D1 foals showed higher (*p* < 0.001) total ketones content than the D2 group (22.80 *vs.* 17.67 AU × 10^4^/g fresh meat, respectively). On the other hand, a similar trend was found in recent studies [54,64], where authors observed that animals finished with commercial concentrates reported higher values than those reared under an extensive production system. Similarly, all singular ketones showed higher values in the D1 group in comparison with the D2 one (*p* < 0.05), apart from 2,3-pentanedione. Among them, acetoin represented the most plentiful compounds in D1 foals, corresponding to 31.24% of the total ketone content. According to several studies [72,73,74], the production of acetoin could be related to the microbial carbohydrate metabolism. Moreover, this volatile compound has a very low odor threshold, being easily perceived by olfactory analyzers, and is characterized by a buttery, cream-like and sweet aroma [75]. Hence, the presence of different contents could affect and generate modifications at several levels in the aromatic profile of the meat [76]. However, this ketone was not detected in previous studies [25,27], investigating the volatile profile of raw foal meat from animals fattened with concentrates; whereas, it was found in fresh lamb meat reared under intensive and extensive systems, but their values were hugely higher than ours [54]. In addition, D1 foals, as commented, showed higher contents of two ketones correlated to lipid degradation, 2-heptanone and butyrolactone [64,77,78,79]. In particular, some authors [77] reported that 2-heptanone could be originated by the oxidation of linoleic acid (C18:2*n*-6). Recent studies [54,80,81], in fact, observed a high presence of this ketone in concentrated-fed ruminants and related this outcome to the elevated content of C18:2*n*-6 in concentrate feeds. For this reason, this volatile compound is defined characteristic of animals fed on commercial concentrates [54,76]. In our study, D1 and D2 groups reported similar values of this fatty acid (*p* > 0.05) (Table 2), probably due its high contents in the concentrates employed both in D1 and D2 (Table 1). Thus, this ketone cannot be considered a direct and appropriate biomarker of the D1 group. However, it could mark the aromatic profile of this group, since several studies [23,82,83] reported that ketones, especially 2-ketones (as 2-heptanone), had a relevant effect/influence on the flavor of meat, owing to their low detection odor threshold and peculiar aroma (as spicy, butter, cheese notes, and ethereal). As regards butyrolactone, researchers found that this lactone is normally formed in ruminants from the corresponding hydroxyl-fatty acids, which are formed in the rumen by the oxidation of oleic (C18:1*n*-9) and linoleic acid (C18:2*n*-6) supplied in the diet [54,79]. In this respect, the abundance of butyrolactone in the D1 group could be especially related to the higher contents in C18:2*n*-6 detected in D1 feeds (Table 1) and reflected also in the fatty acid profile of D1 foals (Table 2). Our data are consistent with those shown by other researchers [64], who reported a higher quantity of this ketone in animals finished with commercial concentrates. Therefore, according to the finishing diet, this family of volatile compounds did not contribute significantly to the total volatile compounds of our samples; in fact, it simply represented the seventh most abundant percentage.

Among the four groups of study, BU-D1 recorded the highest amounts in total ketones (25.26 AU × 10^4^/g fresh meat), followed by JN-D1 (21.10 AU × 10^4^/g fresh meat), BU-D2 (18.80 AU × 10^4^/g fresh meat) and JN-D2 (16.86 AU × 10^4^/g fresh meat). These outcomes reflected what commented above, confirming that the BU breed and D1 treatment favored a major production of ketones compounds. Thus, typical ketone aroma could be detected in BU-D1 foals. Nevertheless, significant interactions between the main fixed factors (breed × finishing diet) were not detected, except for 2,3-pentanedione, acetoin and 5-hexene-3-one.

#### 3.2.6. Esters, Ethers, Furans, Nitrogen Compounds and Sulfur Compounds

Table 4 shows the effect of breed and finishing diet on compounds belonging to the ester family. In this study, six esters were identified in our samples and the total content was shown to be unaffected by breed (*p* > 0.05). In fact, although four out of the six compounds identified presented significant differences (*p* < 0.05), the esters with the majority amounts (vinyl butyrate and caproic acid vinyl ester) reported similar values in both BU and JN foals. Comparing our data with literature, any esters detected in our foals coincided with those found by other authors, studying GM × HB and GM raw foal meat [25,26,27], probably due to the different breeds employed. Additionally, the total ester content occupied 4.22 and 4.95% (for BU and JN foals, respectively) (Figure 1) of the total volatile compounds, corresponding to the fourth most abundant family detected in both breeds. These outcomes disagree with those published by previous studies in raw foal meat [25,26,27], where esters represented the most plentiful compounds.

On the other hand, data showed that the type of diet significantly influenced the total esters content, where the D1 group recorded significantly higher values than the D2 group (*p* < 0.001). As regards the singular substances, vinyl butyrate and caproic acid vinyl ester (the most abundant esters) followed the same trend. Similarly, previous studies [54,64] observed this tendency, indicating that animals fattened with conventional concentrates presented greater ester contents. These differences could be explained by the variability of the fatty acid profile of foals, since esters arise from the esterification of several carboxylic acids in meat [25,26,27,74]. In this regard, as shown in Table 2, D1 and D2 reported significantly different lipid profiles, revealing that 20 out of the 22 fatty acids identified were affected by the type of diet. Thus, these outcomes could justify the behavior of the esters in our study. Moreover, esters are defined as very fragrant compounds [26] which are able to modulate the global flavor owing to their low odor thresholds, imparting fruity notes, especially those composed by short-chain acids [59], while esters formed from long-chain acids provide a slightly fatty odor [84]. However, this class of compounds represented the fourth most abundant family for D1 and D2 groups, considering the total amounts of volatile compounds (Figure 1). Therefore, although the fraction of esters to the total volatile compounds was relatively high (4.97 in D1 foals and 4.24% for D2 foals) (Figure 1), it is complex to affirm that their presence may contribute to the overall aroma of the foal meat.

As regards the four groups of treatment, JN-D1 foals produced the highest amounts of esters, followed by BU-D1 ones, while JN-D2 showed the lowest levels, confirming the data discussed above. In addition, statistical analysis showed the presence of significant interactions among the main factors (breed × finishing diet) in all variables, except for dibutyl sulfate.

With respect to the ether family, only four different compounds were identified in the *longissimus thoracis et lumborum* muscle of foals (Table 4). Breed affected both the total content and each individual ether, showing in all cases significantly higher (*p* < 0.05) values in BU foals, apart from oxirane, tetramethyl-. Nevertheless, despite this marked tendency, this family has not been detected previously in raw foal meat [25,26,27]. A recent study related its presence in meat to possible environmental contamination, since the vapors of certain ethers are employed as fumigants to treat the soil against insects and mites [61]. Thus, their use as biomarkers may be inappropriate. Moreover, considering the ether contribution to the total volatile profile, these substances only represented 0.82 and 0.61% of the total content in BU and JN foals, respectively (Figure 1), being clearly irrelevant in the aromatic profile of foal meat.

Moreover, statistical analysis showed that the type of finishing diet also had a significant effect on the total ether content, recording greater values in the D1 group than in D2 foals (28.23 *vs.* 24.94 AU × 10^4^/g fresh meat, respectively). As regards singular ethers, only two compounds (dimethyl ether and oxirane, tetramethyl-) showed significant differences among groups (*p* < 0.05), where D2 foals presented the lowest values. Our findings are consistent with those published by other authors [54,64], who found that meat from animals finished with concentrates generated higher ether amounts than those reared under an extensive system. However, also in this case, ethers occupied a low percentage of the total volatile compounds detected in both foal groups, occupying the sixth position of the ten families.

Lastly, BU-D1 represented the group with the highest ether content among the four groups, confirming our outcomes. In addition, significant interactions were found among the principal factors (breed × finishing diet) in all compounds, except for methane, oxybis[dichloro-.

Considering the furan family, only two furans were identified in our samples (Table 4). According to statistical analysis, both the total content and individual values were shown to be unaffected by breed (*p* > 0.05), reporting similar values for BU and JN foals. In the same way, the contribution to the volatile profile was almost the same in both breeds, showing a percentage of 0.31 and 0.38% for BU and JN foals, respectively, being the third least abundant family (Figure 1). On the other hand, our data are in contrast with those published by other authors studying foal meat [25,26], where any furans were identified in raw foal meat from GM × HB reared under similar conditions to ours.

Instead, the dietetic regime reported a relevant effect on total furan contents, showing higher (*p* < 0.001) values in the D1 group than the D2 group (17.17 *vs.* 9.56 AU × 10^4^/g fresh meat, respectively). Similarly, this tendency was observed in singular furan contents. Furan, 2-pentyl- especially represented the most abundant furan identified in both diets, showing the lowest (*p* < 0.001) values in D2 foals. These outcomes are in line with those found in the literature [54,64,67]. Concretely, our results could be related to the greater α-tocopherol content of the grass diets (or mainly composed by silage as D2 treatment), since Vasta et al. [60] observed a negative correlation between this antioxidant and the formation of furan, 2-pentyl-. Additionally, this volatile compound is also related to lipid oxidation, in particular to C18:2*n*-6 degradation [22,29]. Our data, however, did not present a direct correspondence with this fatty acid, since both diets recorded similar values, as showed in Table 2. Furans are normally associated to green bean and butter flavors [58]. Nevertheless, considering the percentage represented by these compounds with respect to the total volatiles, furans were the family with the third lowest contribution to the volatility pattern (Figure 1). Thus, furans surely cannot be considered as biomarkers in the aroma of our foals.

Considering the four groups of study, JN-D1 presented the highest values, whereas JN-D2 had the lowest ones, marking the significant differences among the two dietetic regimes previously discussed. Therefore, significant interactions among breed and finishing diet were found, except for furan, 2-ethyl-.

The influence of breed and type of diet on the nitrogen compounds detected in the *longissimus thoracis et lumborum* muscle of foals is showed in Table 4. Four different compounds were identified and their total contents resulted to be significantly (*p* < 0.001) affected by breed, presenting greater values in BU foals than in JN ones (556.99 *vs.* 391.37 AU × 10^4^/g fresh meat, respectively). As regards the individual compounds, breed had a significant (*p* < 0.05) effect on most of them, apart from propane, 2-nitro-. Concretely, 2-propen-1-amine represented the majority substance within this family, and the BU group values almost duplicate those of JN foals (*p* < 0.001).

On the other hand, the type of diet also significantly (*p* < 0.05) influenced the total nitrogen compounds contents, presenting higher values in the D1 group in comparison with the D2 one. In this case, among individual compounds, significant (*p* < 0.05) differences were observed in the minority nitrogen substances (diazene, dimethyl- and propane, 2-nitro); whereas the predominant compounds showed similar (*p* > 0.05) values in both diet groups.

This family represented the second most plentiful of the total volatile compounds in all groups (Figure 1). However, this class of substances has not been previously detected in any volatile profile of raw foal meat [25,26,27].

Considering the four group of studies, BU-D1 presented the highest amounts, while JN-D2 had the lowest values. However, any significant interactions among the main factors (B × D) were observed, expect for diezene, dimethyl- and 2-propanamine.

Regarding the sulfur compounds, only one substance was detected, named carbone disulphide. Table 4 showed that breed significantly (*p* < 0.05) affected carbon disulphide amounts, being higher in JN foals than BU ones (8.49 *vs.* 6.62 × 10^4^/g fresh meat, respectively). According to the literature, this volatile compound could be a product of the enzymatic proteolysis of sulfur-containing amino acids [85], but can be also originated from fungicides used in agriculture [86]. However, there is scarce information about the amino acid profile of both breeds, so it is complicated to compare these results and understand the exact origin of this compound. However, carbon disulphide corresponded to a very low fraction of the total volatile compounds, being the family with the second (JN foals) and the lowest (BU foals) contribution to the volatile profile (Figure 1).

Concerning the diet effect, statistical analysis showed a tendency (*p* < 0.1) among both diets, recording higher values in the D2 group compared with D1 foals (8.48 *vs*. 6.88 × 10^4^/g fresh meat, respectively). This difference is consistent with data previously reported [54,64,65]; in fact, several authors observed that animals with grass-based diets obtained higher contents of sulfur compounds than those finished with concentrates. Vasta and Priolo [65] affirmed that this trend could be explained by the fact that herbage diets can produce an increase in free amino acids. Hence, the formation of sulfur compounds could be favored, since these substances derive from the enzymatic proteolysis of the sulfur-containing amino acids [85], as aforementioned. However, also in this case, the contribution of carbon disulphide on the total volatile contents was minimal, as shown in Figure 1. The four groups of the study showed similar values, except for JN-D2 foals, which presented the highest values of carbon disulphide. However, any significant interactions were observed among the main factors.

As a general conclusion, it is well known that fat content plays an important role both in volatile compounds and in the aroma of meat and meat products [22]. Thus, the volatile compounds derived from lipids are the most important in meat [22,29], and the special property of lipids to solubilize and serve as a reservoir for these volatile compounds also stands out. Therefore, it is expected that meat with a higher fat content has a higher content of volatile compounds, and more specifically of lipid-derived ones. In this study, BU foals (5.01% BU *vs.* 3.79% JN), as well as animals fed with diet 1 (4.88% D1 *vs.* 3.76% D2), had the highest fat content. Therefore, a large part of the results obtained in the present study can be attributed to both the amount and the lipid composition of the meat.

## 4. Conclusions

From the results, we can conclude that the breed and the finishing diet had a significant influence on the fatty acid profile of the *longissimus thoracis et lumborum muscle* from foals. In particular, JN foals reported a slightly higher content of SFA, counterbalanced by significantly greater PUFA amounts. Furthermore, JN samples showed a favorable PUFA/SFA ratio, whereas, considering the other nutritional indices, both breeds presented values complying with the recommendations. On the other hand, the type of finishing diet confirmed its key role on meat lipid composition. Diet 2 was actually demonstrated to strongly ameliorate the fatty acid profile of meat, favoring a reduction of SFA fraction and a significant increase of PUFA contents. Concretely, D2 foals reported greater *n*-3 values, principally owing to their elevated concentrations of α-linolenic acid and LC *n*-3 FA. As a result, the combination of silage and organic feed also positively affected the nutritional parameters of this group, recording the best values according to the health guidelines.

Similarly, volatile profile was affected by the breed but especially by the kind of diet. BU foals recorded higher total content of volatile compounds, being predominant in half of the families identified (total hydrocarbons, acids, ketones, ethers, nitrogen compounds), whereas similar values were observed in alcohol, aldehyde, ester and furan total amounts in both groups. On the other hand, the type of finishing diet strongly affected the total amounts of the families detected and the level of most of their singular compounds, showing greater values in D1 foals, apart from the family of sulfur compounds.

On the whole, among the first five most plentiful families identified, there are classes of compounds strictly related to meat lipid oxidation and its fatty acid composition, such as hydrocarbons, aldehydes, esters and alcohols. In this sense, diet 2 demonstrated a chief role in this study, since its “ingredients” (silage and organic feed) and composition favored not only the improvement of the lipid profile and nutritional indices of foal meat, but also decreased the generation of volatile compounds associated with lipid oxidation, and minimized off-flavors. This last action, actually, may make the difference in the aromatic perception and, as a consequence, in the sensorial acceptability of this meat.

In closing, further studies are necessary to characterize the “dietetic” and volatile profile of the meat from these Navarre autochthonous foals. Otherwise, considering the limited information about these parameters in fresh foal meat, especially in the case of the Jaca Navarra breed, this work could be considered a pioneer study in order to extend our knowledge about these valuable endangered foal breeds.

## Figures and Tables

**Table 1:** Chemical composition (expressed as percentage) and fatty acid profile (expressed as g/100 g of fatty acids) of oats, concentrates, straw and silage allocated to the foals.

	Oats	Starter Feed	Finisher Feed	Straw	Silage	Organic Feed
**Chemical composition (%)**						
Fat	5.58	3.40	6.00	1.95	3.71	4.80
Protein	8.78	12.90	12.80	2.00	16.22	8.50
Ash	2.59	8.50	4.50	3.88	8.81	5.00
**Fatty acids (g/100 g of fatty acids)**						
C8:0	0.02	0.17	0.00	0.91	0.00	0.00
C10:0	0.01	0.17	0.04	0.78	0.13	0.00
C12:0	0.02	2.17	0.18	0.98	0.14	0.01
C14:0	0.19	1.11	0.73	3.74	0.20	0.14
C15:0	0.01	0.09	0.07	0.50	0.12	0.01
C16:0	15.96	22.58	22.00	29.25	16.98	14.05
C16:1*n*-7	0.19	0.22	0.80	0.74	0.15	0.14
C17:0	0.06	0.15	0.18	0.21	0.32	0.10
C18:0	1.21	2.67	3.83	6.39	1.52	2.42
9*t*-C18:1	0.02	0.03	0.13	0.51	0.20	0.00
11*t*-C18:1	0.00	0.00	0.23	0.00	0.00	0.00
C18:1*n*-9	37.74	26.08	34.88	14.53	2.73	28.90
C18:1*n*-7	1.25	1.20	1.74	0.97	0.46	1.26
C18:2*n*-6	40.09	36.80	31.26	19.64	17.12	47.24
C18:3*n*-3	1.35	4.85	2.22	13.10	56.63	3.98
C20:0	0.15	0.29	0.20	1.73	0.37	0.29
C20:1*n*-9	0.83	0.47	0.57	0.00	0.19	0.55
C21:0	0.02	0.03	0.02	0.69	0.26	0.04
C22:0	0.13	0.23	0.00	2.10	0.61	0.00
C20:5*n*-3 (EPA)	0.00	0.00	0.14	0.00	0.00	0.27
C22:1*n*-9	0.51	0.17	0.22	0.31	0.10	0.26
C22:2*n*-6	0.01	0.04	0.00	0.00	0.77	0.00
C24:0	0.09	0.23	0.10	2.09	0.98	0.17
C22:6*n*-3 (DHA)	0.08	0.08	0.11	0.00	0.00	0.07
SFA	17.90	29.98	27.37	50.20	21.63	17.28
MUFA	40.55	28.18	38.60	17.06	3.83	31.11
PUFA	41.55	41.84	34.04	32.75	74.54	51.62

EPA: eicosapentaenoic acid; DHA: docosahexaenoic acid; SFA: saturated fatty acids; MUFA: monounsaturated fatty acids; PUFA: polyunsaturated fatty acids.

**Table 2:** Effect of breed and finishing diet on fatty acids profile (expressed as g/100 g of fat) of *longissimus thoracis et lumborum* muscle of foals.

	JN		BU			Sig.		
Fatty Acids	D1	D2	D1	D2	SEM	B	D	B × D
C10:0	0.08 ^b^	0.05 ^a^	0.08 ^b^	0.05 ^a^	0.002	ns	***	ns
C12:0	0.18 ^c^	0.12 ^a^	0.20 ^c^	0.14 ^b^	0.006	**	***	ns
C14:0	3.29 ^b^	2.49 ^a^	3.50 ^b^	2.64 ^a^	0.078	ns	***	ns
C14:1*n*-5	0.38 ^b^	0.22 ^a^	0.50 ^c^	0.27 ^a^	0.018	***	***	ns
C15:0	0.23 ^b^	0.15 ^a^	0.24 ^b^	0.14 ^a^	0.007	ns	***	ns
C16:0	26.48 ^b^	25.32 ^ab^	25.63 ^ab^	24.73 ^a^	0.245	ns	*	ns
C16:1*n*-7	6.84 ^b^	5.32 ^a^	8.21 ^c^	5.58 ^a^	0.218	*	***	ns
C17:0	0.26 ^c^	0.22 ^b^	0.25 ^c^	0.20 ^a^	0.005	*	***	ns
C18:0	3.84 ^c^	4.01 ^c^	3.10 ^a^	3.49 ^b^	0.069	***	**	ns
9*t*-C18:1	0.13 ^c^	0.12 ^b^	0.11 ^b^	0.10 ^a^	0.002	***	***	ns
C18:1*n*-9	28.73 ^bc^	26.00 ^a^	29.56 ^c^	26.96 ^ab^	0.391	ns	***	ns
C18:1*n*-7	1.63 ^b^	1.35 ^a^	1.84 ^c^	1.33 ^a^	0.038	*	***	*
C18:2*n*-6	10.24 ^b^	9.84 ^b^	7.42 ^a^	8.28 ^a^	0.256	***	ns	ns
C18:3*n*-3	3.16 ^a^	4.41 ^b^	2.64 ^a^	4.51 ^b^	0.174	ns	***	ns
C20:1*n*-9	0.31 ^b^	0.28 ^ab^	0.30 ^b^	0.25 ^a^	0.007	ns	**	ns
C20:2*n*-6	0.19 ^b^	0.19 ^b^	0.13 ^a^	0.15 ^a^	0.006	***	ns	ns
C20:3*n*-6	0.16 ^b^	0.20 ^c^	0.11 ^a^	0.15 ^b^	0.006	***	***	ns
C20:4*n*-6	0.68 ^bc^	0.80 ^c^	0.40 ^a^	0.56 ^b^	0.030	***	**	ns
C20:3*n*-3	0.13 ^b^	0.19 ^d^	0.10 ^a^	0.16 ^c^	0.006	**	***	ns
C20:5*n*-3 (EPA)	0.05 ^a^	0.09 ^b^	0.05 ^a^	0.08 ^b^	0.004	ns	***	ns
C22:5*n*-3 (DPA)	0.29 ^b^	0.48 ^d^	0.21 ^a^	0.39 ^c^	0.020	**	***	ns
C22:6*n*-3 (DHA)	0.06 ^ab^	0.10 ^c^	0.06 ^a^	0.09 ^bc^	0.006	ns	**	ns
SFA	34.45 ^b^	32.45 ^a^	33.08 ^ab^	31.46 ^a^	0.311	*	**	ns
MUFA	38.04 ^b^	33.30 ^a^	40.53 ^b^	34.49 ^a^	0.613	ns	***	ns
PUFA	14.99 ^b^	16.32 ^b^	11.14 ^a^	14.38 ^b^	0.419	***	**	ns
*n*-3	3.69 ^a^	5.27 ^b^	3.05 ^a^	5.23 ^b^	0.197	ns	***	ns
*n*-6	11.29 ^b^	11.05 ^b^	8.08 ^a^	9.15 ^a^	0.289	***	ns	ns
LC *n*-3	0.40 ^a^	0.67 ^c^	0.31 ^a^	0.56 ^b^	0.028	*	***	ns
*n*-6/*n*-3	3.12 ^c^	2.17 ^b^	2.77 ^c^	1.77 ^a^	0.102	**	***	ns
PUFA/SFA	0.44 ^b^	0.51 ^b^	0.34 ^a^	0.46 ^b^	0.015	**	***	ns
TI	0.94 ^b^	0.84 ^a^	0.95 ^b^	0.81 ^a^	0.015	ns	***	ns
AI	0.75 ^ab^	0.71 ^a^	0.77 ^b^	0.73 ^a^	0.008	ns	**	ns
h/H	1.50 ^a^	1.54 ^a^	1.44 ^a^	1.53 ^a^	0.017	ns	ns	ns

^a–d^ Mean values in the same row (corresponding to the same parameter) with different letters differ significantly (*p* < 0.05; Duncan test); SEM: standard error of the mean; Sig.: significance: *** (*p* < 0.001), ** (*p* < 0.01), * (*p* < 0.05), ns. (not significant); D1 (Diet 1) = conventional concentrate + straw, D2 (Diet 2) = silage + organic concentrate; B = breed; D = finishing diet. SFA: saturated fatty acids; MUFA: monounsaturated fatty acids; PUFA: polyunsaturated fatty acids; *n*-3: omega-3; *n*-6: omega-6; LC *n*-3: long chain omega-3; TI: thrombogenic index; AI: atherogenic index; h/H: hypo/hypercholesterolemic fatty acids ratio.

**Table 3:** Effect of breed and finishing diet on hydrocarbons, acids and alcohols (expressed as volatile AU x 10^4^/g fresh meat) of *longissimus thoracis et lumborum* muscle of foals.

Volatile Compounds	LRI	*m/z*	JN		BU			Sig.		
			D1	D2	D1	D2	SEM	B	D	B × D
Isobutane	488	57	0.24 ^ab^	0.19 ^a^	0.30 ^b^	0.22 ^ab^	0.015	ns	*	ns
Pentane, 2-methyl-	516	71	0.25 ^a^	0.21 ^a^	0.51 ^b^	0.58 ^b^	0.029	***	ns	ns
Pentane, 3-methyl-	525	57	2.24 ^b^	1.41 ^a^	3.67 ^c^	3.30 ^c^	0.173	***	*	ns
Pentane	500	43	1.30 ^b^	0.87 ^a^	1.14 ^ab^	0.92 ^a^	0.060	ns	**	ns
Cyclopentane, methyl-	564	56	1.47 ^a^	1.13 ^a^	3.13 ^b^	2.75 ^b^	0.160	***	ns	ns
Bicyclo[3.2.0]hepta-2,6-diene	790	91	2670 ^b^	2210 ^a^	2981 ^b^	2801 ^b^	86.640	**	*	ns
Octane	800	85	1.95 ^ab^	1.63 ^a^	2.18 ^b^	1.44 ^a^	0.091	ns	**	ns
Nonane	900	57	0.65 ^b^	0.43 ^a^	0.76 ^b^	0.75 ^b^	0.033	***	*	ns
Cyclopentane, 1,1-dimethyl-	927	69	0.70 ^c^	0.34 ^a^	0.59 ^bc^	0.50 ^b^	0.032	ns	***	*
Cyclopropane	1041	55	25.64 ^b^	16.03 ^a^	19.75 ^a^	20.20 ^a^	1.027	ns	*	**
Undecane, 5,5-dimethyl-	1069	71	0.27 ^a^	1.53 ^b^	0.13 ^a^	0.07 ^a^	0.102	***	***	***
Cyclobutane, butyl-	1119	84	0.86 ^b^	0.36 ^a^	0.74 ^b^	0.40 ^a^	0.039	ns	***	ns
Dodecane	1200	71	0.20 ^b^	0.40 ^c^	0.22 ^b^	0.07 ^a^	0.023	***	ns	***
**Total lineal hydrocarbons**			4.35 ^b^	3.53 ^a^	4.60 ^b^	3.40 ^a^	0.135	ns	***	ns
**Total branched hydrocarbons**			2.76 ^a^	3.15 ^a^	4.31 ^b^	3.95 ^b^	0.156	***	ns	ns
**Total cyclic hydrocarbons**			2698 ^b^	2228 ^a^	3006 ^b^	2825 ^b^	86.732	**	*	ns
**Total hydrocarbons**			2705 ^b^	2235 ^a^	3015 ^b^	2832 ^b^	86.831	**	*	ns
Butanoic acid, 4-hydroxy-	1038	86	4.49 ^b^	3.98 ^b^	4.20 ^b^	2.95 ^a^	0.170	*	**	ns
Hexanoic acid	1074	60	5.73 ^b^	0.76 ^a^	9.46 ^c^	4.36 ^b^	0.524	***	***	ns
2-Furancarboxylic acid, tetrahydro-3-methyl-5-oxo-	1131	99	1.44 ^c^	0.45 ^a^	1.81 ^d^	1.02 ^b^	0.092	***	***	**ns**
**Total acids**			11.65 ^c^	5.20 ^a^	15.48 ^d^	8.33 ^b^	0.642	***	***	ns
Glycidol	472	44	3.52 ^b^	1.78 ^a^	2.93 ^b^	2.05 ^a^	0.165	ns	***	ns
Cyclobutanemethanol	716	57	19.47 ^b^	11.47 ^a^	15.75 ^ab^	19.01 ^b^	0.942	ns	ns	**
1-Pentanol	834	70	46.05 ^c^	26.67 ^a^	35.05 ^b^	29.94 ^ab^	1.750	ns	***	*
Prenol	843	71	0.72 ^b^	0.54 ^a^	0.55 ^a^	0.68 ^b^	0.024	ns	ns	**
Cis-2-Pentenol	843	57	2.15 ^b^	1.57 ^a^	2.28 ^b^	2.48 ^b^	0.098	**	ns	*
2,3-Butanediol	906	45	0.68 ^a^	0.70 ^a^	1.55 ^b^	0.85 ^a^	0.071	***	**	**
1-Hexanol	943	56	10.71 ^c^	4.94 ^a^	11.62 ^c^	7.60 ^b^	0.511	**	***	ns
5-Methyl-1-heptanol	1029	83	6.05 ^b^	2.94 ^a^	5.60 ^b^	4.93 ^b^	0.283	**ns**	***	**
1-Heptanol	1036	70	3.36 ^b^	1.79 ^a^	3.54 ^b^	1.92 ^a^	0.165	ns	***	ns
1-Octen-3-ol	1042	57	103.5 ^b^	69.38 ^a^	73.66 ^a^	71.70 ^a^	3.599	*	**	*
Diethylene glycol	1080	45	1.19 ^b^	0.39 ^a^	1.03 ^b^	0.25 ^a^	0.080	ns	***	ns
2-Ethyl-1-hexanol	1085	57	1.06 ^b^	0.71 ^a^	1.32 ^c^	1.08 ^b^	0.043	***	***	**ns**
1-Octanol	1119	56	1.26 ^b^	0.68 ^a^	1.20 ^b^	0.66 ^a^	0.058	ns	***	ns
6-Undecanol	1156	83	1.78 ^b^	0.45 ^a^	2.52 ^c^	0.54 ^a^	0.157	*	***	ns
**Total alcohols**			201.5 ^c^	124.0 ^a^	158.6 ^b^	143.7 ^ab^	6.285	ns	***	**

^a–d^ Mean values in the same row (corresponding to the same parameter) with different letters differ significantly (*p* < 0.05; Duncan test); SEM: standard error of the mean; Sig.: significance: *** (*p* < 0.001), ** (*p* < 0.01), * (*p* < 0.05), ns. (not significant); D1 (Diet 1) = conventional concentrate + straw, D2 (Diet 2) = silage + organic concentrate; B = breed; D = finishing diet; LRI: lineal retention index calculated for the DB-624 capillary column (30 m × 0.25 mm id, 1.4 μm film thickness; J&W Scientific, Folsom, CA, USA), installed on a gas chromatograph equipped with a mass selective detector; *m/z*: quantification ion.

**Table 4:** Effect of breed and finishing diet on aldehydes, ketones, esters, ethers, furans, nitrogen and sulfur volatile compounds (expressed as volatile AU × 10^4^/g fresh meat) of *longissimus thoracis et lumborum* muscle of foals.

Volatile Compounds	LRI	*m/z*	JN		BU			Sig.		
			D1	D2	D1	D2	SEM	B	D	B × D
Propanal	499	58	9.70 ^b^	5.42 ^a^	10.29 ^b^	11.74 ^b^	0.531	***	ns	**
2-Propynal	537	53	7.47 ^a^	7.41 ^a^	13.43 ^b^	12.60 ^b^	0.583	***	ns	ns
Pentanal	714	57	15.66 ^b^	8.05 ^a^	11.07 ^a^	15.28 ^b^	0.802	ns	ns	***
Hexanal	853	56	294.0 ^c^	140.0 ^a^	207.1 ^b^	198.4 ^ab^	13.513	ns	***	**
Heptanal	963	70	7.83 ^b^	2.07 ^a^	8.91 ^b^	3.78 ^a^	0.517	*	***	ns
2-Heptenal, (Z)-	1029	83	6.70 ^c^	3.29 ^a^	4.27 ^ab^	4.90 ^b^	0.296	ns	**	***
Octanal	1057	84	1.98 ^c^	0.80 ^a^	1.98 ^c^	1.27 ^b^	0.107	ns	***	ns
2-Octenal, (E)-	1116	83	2.05 ^c^	0.99 ^a^	1.88 ^bc^	1.47 ^ab^	0.106	ns	***	ns
Nonanal	1141	98	4.21 ^c^	1.78 ^a^	3.69 ^bc^	3.03 ^b^	0.198	ns	***	**
**Total aldehydes**			349.6 ^c^	169.9 ^a^	262.6 ^b^	252.4 ^b^	15.017	ns	***	***
2,3-Pentanedione	722	100	5.28 ^ab^	4.08 ^a^	5.80 ^b^	8.29 ^c^	0.309	***	**ns**	***
Acetoin	776	45	5.41 ^a^	5.74 ^a^	9.60 ^b^	4.07 ^a^	0.438	ns	***	***
2-Heptanone	956	58	3.03 ^b^	1.35 ^a^	2.73 ^b^	1.55 ^a^	0.167	ns	***	ns
Butyrolactone	1038	86	4.49 ^b^	3.98 ^b^	4.20 ^b^	2.95 ^a^	0.170	*	**	ns
5-Hexen-3-one	1075	69	0.55 ^b^	0.41 ^b^	0.87 ^c^	0.22 ^a^	0.039	ns	***	***
3-Octen-2-one	1099	111	2.34 ^b^	1.30 ^a^	2.05 ^b^	1.73 ^ab^	0.122	ns	**	ns
**Total ketones**			21.10 ^b^	16.86 ^a^	25.26 ^c^	18.80 ^ab^	0.696	**	***	ns
Dibutyl sulphate	525	56	1.65 ^a^	1.24 ^a^	3.35 ^b^	2.91 ^b^	0.163	***	ns	ns
Vinyl butyrate	1041	71	102.55 ^b^	60.71 ^a^	77.61 ^a^	77.90 ^a^	3.717	ns	**	**
Caproic acid, vinyl ester	1041	99	115.3 ^b^	61.33 ^a^	93.08 ^b^	89.63 ^b^	5.121	ns	**	**
Glycerol 1,2-diacetate	1333	145	0.37 ^b^	0.26 ^a^	0.34 ^ab^	0.55 ^c^	0.021	***	ns	***
Butyl isobutyrate	1355	89	0.62 ^b^	0.16 ^a^	0.24 ^a^	0.20 ^a^	0.036	***	***	***
2,2,4-Trimethyl-1,3-pentanediol diisobutyrate	1439	71	4.45 ^b^	2.90 ^a^	2.11 ^a^	2.38 ^a^	0.206	***	ns	**
**Total esters**			224.9 ^c^	126.5 ^a^	176.7 ^b^	173.5 ^b^	8.673	ns	***	**
Dimethyl ether	491	45	5.80 ^a^	7.03 ^a^	14.52 ^c^	9.53 ^b^	0.618	***	*	***
Tetrahydrofuran	587	72	2.69 ^a^	6.08 ^b^	8.18 ^c^	4.69 ^b^	0.433	**	ns	***
Methane, oxybis[dichloro-	591	83	10.45 ^ab^	9.04 ^a^	16.68 ^c^	13.59 ^bc^	0.737	***	ns	ns
Oxirane, tetramethyl-	883	59	1.21 ^c^	0.45 ^a^	0.73 ^b^	0.42 ^a^	0.064	**	***	*
**Total ethers**			20.15 ^a^	22.60 ^a^	40.11 ^c^	28.23 ^b^	1.408	***	*	***
Furan, 2-ethyl-	688	81	2.82 ^b^	1.86 ^a^	2.90 ^b^	2.60 ^ab^	0.155	ns	*	ns
Furan, 2-pentyl-	1027	81	15.79 ^c^	6.72 ^a^	12.19 ^b^	8.33 ^a^	0.775	ns	***	*
**Total furans**			18.61 ^b^	8.58 ^a^	15.08 ^b^	10.94 ^a^	0.893	ns	***	*
Diazene, dimethyl-	502	58	2.95 ^b^	4.61 ^c^	1.91 ^a^	2.08 ^a^	0.205	***	**	**
2-Propen-1-amine	535	56	235.3 ^a^	154.1 ^a^	390.2 ^b^	367.7 ^b^	20.813	***	ns	ns
2-Propanamine	714	58	7.02 ^b^	3.02 ^a^	3.91 ^a^	3.53 ^a^	0.380	*	***	**
Propane, 2-nitro-	1041	43	218.0 ^b^	162.7 ^a^	172.6 ^ab^	173.0 ^ab^	8.762	ns	ns	ns
**Total nitrogen compounds**			463.3 ^b^	324.5 ^a^	568.7 ^b^	546.4 ^b^	22.754	***	*	ns
Carbon disulfide	506	76	7.31 ^a^	9.59 ^b^	6.27 ^a^	6.93 ^a^	0.412	*	ns	ns
**Total sulfur compounds**			7.31 ^a^	9.59 ^b^	6.27 ^a^	6.93 ^a^	0.412	*	ns	ns
**Total volatile compounds**			4024 ^b^	3043 ^a^	4284 ^b^	4022 ^b^	109.963	***	***	*

^a–c^ Mean values in the same row (corresponding to the same parameter) with different letters differ significantly (*p* < 0.05; Duncan test); SEM: standard error of the mean; Sig.: significance: *** (*p* < 0.001), ** (*p* < 0.01), * (*p* < 0.05), ns. (not significant); D1 (Diet 1) = conventional concentrate + straw, D2 (Diet 2) = silage + organic concentrate; B = breed; D = finishing diet; LRI: lineal retention index calculated for the DB-624 capillary column (30 m × 0.25 mm id, 1.4 μm film thickness; J&W Scientific, Folsom, CA, USA) installed on a gas chromatograph equipped with a mass selective detector; *m/z*: quantification ion.

## Data Availability

All data are presented in the manuscript.
